# Broadening Boundaries and Deepening Understanding of/Within Public Health

**DOI:** 10.3390/ijerph22091376

**Published:** 2025-09-01

**Authors:** Paul R. Ward

**Affiliations:** Centre for Public Health, Equity and Human Flourishing, Torrens University Australia, Adelaide 5000, Australia; paul.ward@torrens.edu.au

As the Editor-in-Chief of the *International Journal of Environmental Research and Public Health* (*IJERPH*) [1], I am proud to reaffirm our journal’s commitment to advancing multidisciplinary research that promotes health and wellbeing for all. Our plan is to address critical public health and environmental topics and strengthen open scientific discussions.

Let us address the elephant in the room. As an academic in public health, I have heard numerous comments about *IJERPH* over the last couple of years, linked to the delisting of the journal by Web of Science (WoS) in 2023. To be clear and to dispel on-going rumours and concerns by public health academics, *IJEPRH* was delisted because there was concern by the WoS that some of the topics in some of the papers did not fit the main title of the journal and were not “public health and environmental health” enough to fit their definition of “public health and environmental health”. Since that decision by WoS, *IJERPH* has developed clearer guidelines on what constitutes “public health and environmental health” and has put that into place in order to make initial decisions before papers go for review (see Table 1 for weblinks to various processes in place concerning integrity and transparency in publishing at *IJERPH*). An application has been made by *IJERPH* to be relisted on WoS.

Please also remember that IJERPH has remained in Scopus, where, with a 2024 CiteScore of 8.5, it is a Q1 journal in three different research categories (ranked 50/687 for “Public Health, Environmental and Occupational Health”, ranked 27/148 for “Health, Toxicology and Mutagenesis”, and ranked 37/176 for “Pollution”). *IJERPH* has also continued to receive strong support from the global academic community. We have been endorsed by a diverse and international group of scholars, with authors representing over 151 countries/regions across all 6 continents (from 16 March 2023 to 31 August 2025). Furthermore, our Editorial Board Members, hailing from 33 countries/regions, have been instrumental in this effort. As of 31 August 2025, published papers in *IJERPH* have been highlighted over 49,400 times in prominent news outlets such as Forbes, CNN News, The Washington Post, BBC News, National Geographic, and The Guardian. *IJERPH* is also ranked #1 in the 2025 Google Scholar Metrics in the category of Public Health. In short, *IJERPH* is a leading international journal in public health, and my aim is to consolidate this strength and broaden our horizons into new or emerging fields of public health, including the commercial determinants of health and planetary health.

Public health is no longer a discipline confined to traditional boundaries; it is a dynamic field shaped by a wide spectrum of interconnected biological, environmental, behavioural, social, economic, political, and commercial determinants of health. *IJERPH* aims to serve as a platform that brings together diverse scientific communities, including social, behavioural and political scientists, epidemiologists, environmental researchers, program managers, and policymakers, to foster a more holistic understanding of public health.

We believe that environmental research must extend beyond ecological studies alone. Instead, it should encompass the complex interplay between individuals and their physical, emotional, commercial and social lifeworlds. Whether exploring urban design, air quality, social cohesion, or behavioural interventions, we welcome studies that offer insight into how these factors affect human health and wellbeing outcomes.

*IJERPH* expects all submissions to explicitly connect their findings to public health and/or health promotion. Rigorous methodology, detailed reporting, and reproducibility are core principles we uphold. Authors are encouraged to provide comprehensive experimental and theoretical data that deepen our understanding and support real-world application.

We publish original research, critical reviews, and short communications that contribute to the advancement of public health knowledge. While we welcome studies focused on regional health issues, these should be positioned within a broader, global context to ensure their wider relevance.

It is important to note that clinical studies that do not clearly align with the scope of our journal will not be considered.

Together, let us continue expanding the scope of public health to embrace its full complexity—and, in doing so, contribute meaningfully to healthier societies worldwide.

## Figures and Tables

**Table 1:** Processes focused on promoting publishing integrity and transparency.

Title	Link	Reference
Rigorous and transparent peer review process	https://www.mdpi.com/editorial_process	[2]
Editorial Independence	https://www.mdpi.com/journal/ijerph/instructions#independence	[3]
Research and Publication Ethics	https://www.mdpi.com/journal/ijerph/instructions#ethics	[4]
Research Data Policies	https://www.mdpi.com/journal/ijerph/instructions#suppmaterials	[5]
Authorship	https://www.mdpi.com/journal/ijerph/instructions#authorship	[6]
Conflicts of Interest	https://www.mdpi.com/journal/ijerph/instructions#conflict	[7]

## References

[1] *International Journal of Environmental Research and Public Health (IJERPH)* Home Page. https://www.mdpi.com/journal/ijerph.

[2] Rigorous and Transparent Peer Review Process. https://www.mdpi.com/editorial_process.

[3] Editorial Independence. https://www.mdpi.com/journal/ijerph/instructions#independence.

[4] Research and Publication Ethics. https://www.mdpi.com/journal/ijerph/instructions#ethics.

[5] Research Data Policies. https://www.mdpi.com/journal/ijerph/instructions#suppmaterials.

[6] Authorship. https://www.mdpi.com/journal/ijerph/instructions#authorship.

[7] Conflicts of Interest. https://www.mdpi.com/journal/ijerph/instructions#conflict.

